# Growth, current size and the role of the 'reversal paradox' in the foetal origins of adult disease: an illustration using vector geometry

**DOI:** 10.1186/1742-5573-3-9

**Published:** 2006-08-02

**Authors:** Yu-Kang Tu, George TH Ellison, Mark S Gilthorpe

**Affiliations:** 1 Biostatistics Unit, Centre for Epidemiology & Biostatistics, University of Leeds, 30/32 Hyde Terrace, Leeds, LS2 9LN, UK; 2 Leeds Dental Institute, University of Leeds, Clarendon Road, Leeds, LS2 9LU, UK; 3 St George's – University of London, Cranmer Terrace, London SW17 0RE, UK

## Abstract

### Background

Numerous studies have reported inverse associations between birth weight and a range of diseases in later life. These have led to the development of the 'foetal origins of adult disease hypothesis'. However, many such studies have only been able to demonstrate a statistically significant association between birth weight and disease in later life by adjusting for current size. This has been interpreted as evidence that the impact of low birth weight on subsequent disease is somehow dependent on subsequent weight gain, and has led to a broadening of the hypothesis into the 'developmental origins of health and disease'. Unfortunately, much of the epidemiological evidence used for both of these interpretations is prone to a statistical artefact known as the 'reversal paradox'. The aim of this paper is to illustrate why, using vector geometry.

## Materials and methods

This paper introduces the key concepts of vector geometry as applied to multiple regression analysis. This approach is then used to illustrate the similar statistical problems encountered when adjusting for current size or growth when exploring the association between birth weight and disease in later life.

## Results

Geometrically, the three covariates – birth size, growth, and current size – span only 2-dimensional space. Regressing disease in later life (i.e. the outcome variable) on any two of these covariates equates to projecting the disease variable onto the plane spanned by the three covariate vectors. The three possible regression models – where any two covariates are considered – are therefore equivalent and yield exactly the same model fit (*R*^2^).

## Conclusion

Vector geometry illustrates why it is impossible to differentiate between the effects of growth from the effects of current size in studies exploring the relationship between size at birth and subsequent disease. For similar reasons, it is impossible to differentiate between the effects of growth and the effects of birth weight. Assessing the 'independent' impact of growth on later disease by adjusting for either birth weight or current size is therefore illusory.

## Background

Numerous studies over the past two decades have found inverse associations between birth weight and a range of chronic diseases – associations which gave rise to the 'foetal origins of adult disease hypothesis'. This argues that under-nutrition or growth retardation *in utero *can have adverse long-term effects on the development of vital organ systems, thereby increasing the risk of a range of metabolic and related disorders such as: hypertension [1]; diabetes [2]; arteriosclerosis [3]; and obesity [4]. However, many such studies have only been able to demonstrate a statistically significant association between birth weight and disease in later life by adjusting for current size [5]. This has been interpreted as evidence that the impact of low birth weight on subsequent disease is somehow dependent on subsequent weight gain, and has led to a broadening of the hypothesis into the 'developmental origins of health and disease' (DOHaD) [6].

Two mechanisms have been postulated to explain the impact of current size on the relation between birth weight and disease in later life. On the one hand, some researchers argue that current size helps to distinguish between those individuals who are genetically small and essentially healthy at birth (i.e. those who remain relatively small in later life), and those who are small at birth as a result of intrauterine growth retardation (i.e. those who subsequently attain a normal or above normal body size, given better conditions for postnatal growth) [7]. On the other hand, other researchers argue that intrauterine conditions leading to growth retardation and low birth weight can elicit permanent yet adaptive physiological responses that are intended to prepare the foetus for a postnatal environment in which nutritional resources are scarce and growth is compromised [8]. In this second mechanism, low birth weight babies who subsequently experience better than expected conditions for postnatal growth are thought to be ill-adapted to cope with normal or excessive nutrition and, as a result, have an increased risk of metabolic and related disorders [8]. Both mechanisms appear plausible, and it is feasible that both might operate at the same time, although the first focuses on developmental damage to organ systems as a result of intrauterine growth retardation, while the latter suggests that its physiological effects are only maladaptive in postnatal environments where growth is no longer compromised.

To help establish the relative importance of pre- and post-natal events on disease in later life, Lucas et al. [9] proposed that four analytical models should be used to establish the role of size at birth, current size and the interaction between the two. However, some researchers have recently questioned the validity of this approach, arguing that it might be inappropriate to adjust for current body size [5], and that testing the interaction between size at birth and current size is equivalent to testing the multivariate normality of birth size, current size and the disease outcome [10]. Our previous studies have confirmed that such adjustments can create a statistical artefact known as the 'reversal paradox' – perhaps better known as 'Simpson's paradox' in the analysis of categorical data [11]. Some researchers might assume that focussing on postnatal weight gain gets around this problem, particularly in studies of children where higher than average postnatal growth amongst low birth weight infants is often interpreted as 'catch-up growth' – a pattern of compensatory growth exhibited by those who have experienced growth retardation *in utero *but are subsequently able to recover what is presumed to be their 'intended' growth trajectory.

In fact, much of the epidemiological evidence used to support a focus on weight gain rather than current weight to explore the 'DOHaD' is based on similarly questionable statistical models – the only difference being in their interpretation. For example, for studies examining systolic blood pressure as the health outcome of interest, most only find a statistically significant inverse relationship with birth weight after adjustment for current weight or body mass index [5]. When there is no adjustment for one or more measures of current body size, the relation between birth weight and blood pressure is substantially reduced and is often not statistically significant [12,13]. For those researchers interested in growth rather than attained size, the statistical effect of adjusting for current weight seems to indicate that there is an interaction between birth weight and current body weight, and that it is more likely to be postnatal growth than size at birth that is relevant to health in later life. This is because the relation between birth weight and blood pressure is substantially weaker without adjustment for current weight [12,13]. In practice this proves to be simply an alternative interpretation of the same, ambiguous statistical relationship – an issue the present study sets out to address using vector geometry to illustrate how focusing on current size or growth, and their associated interpretations, are equally problematic. This is because both scenarios use similar statistical models which are prone to the same statistical artefact, even though they arrive at very different conclusions. Moreover, the present study aims to show that although growth appears to have a larger impact, this cannot be statistically differentiated from that of current size.

To this end, we begin with a concise introduction to vector geometry and use this to illustrate the multiple regression analyses commonly used to explore the foetal origins of adult disease hypothesis. We then demonstrate that the common practice of regressing disease outcomes on birth size and current size does not address the question of whether growth has a greater impact than birth size or current size. For this illustration we use adult systolic blood pressure (*BP*) as the outcome, with birth weight (*BW*), and current weight (*CW*) as potential covariates. A fourth covariate, weight gain (*WG*), is defined as the difference between current weight and birth weight (*CW *- *BW*) and for simplicity, all four variables are treated as continuous. For those interested in a fuller explanation of the basic geometric tools involved, these have been summarised in the Appendix.

## Vector geometry, correlation and regression

### Representation of variables as vectors

Vector geometry is a very useful tool for providing non-statisticians with an intuitive understanding of statistical theory, such as correlation and regression [14,15]. We use vector geometry to illustrate 'simple' (one covariate) and 'multiple' (two or more covariates) regression analyses. To do this, we switch from the more familiar domain of 'variable space' to the less familiar domain of 'subject space'. In variable space, two variables are represented within a plane by a scatter plot, whereas in subject space the same two variables are represented within a plane by two scaled vectors with lengths equal to the standard deviation (SD) of their corresponding variables. The number of dimensions needed to represent variables in subject space is no greater than the number of variables. Although it is impossible to visualize more than three dimensions, only two dimensions are needed to illustrate correlation and simple regression, and only three dimensions are required to illustrate multiple regression.

### Correlation and simple regression

**Figure 1:**
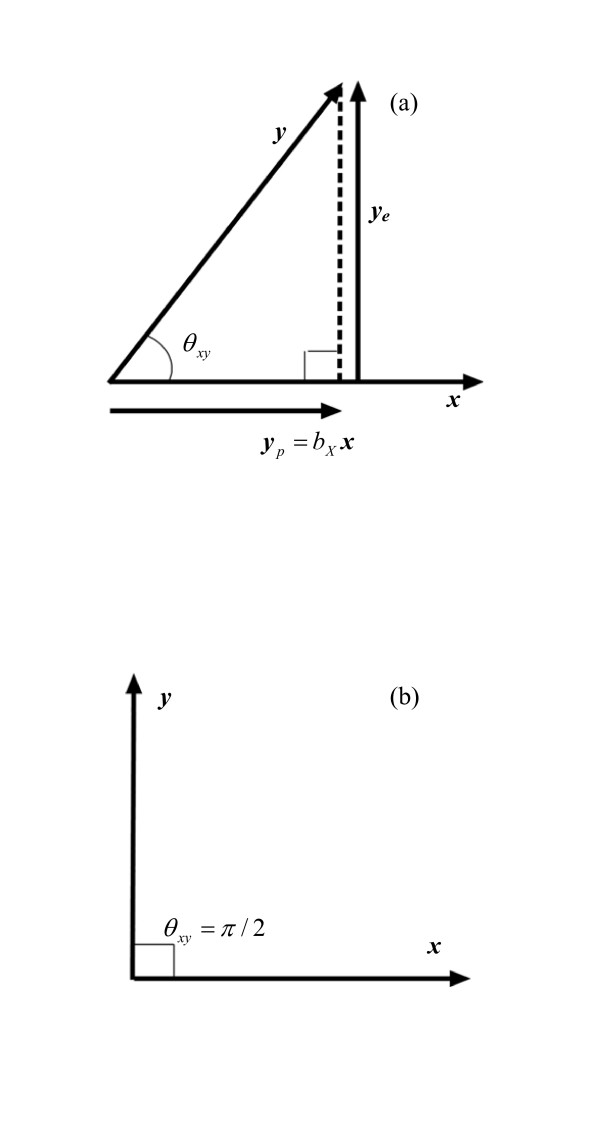
(a) The correlation between variables *Y *and *X *(ρ_*XY*_) is the cosine of θ_*xy*_, the angle between vectors ***x ***and ***y***; the projection of ***y ***on ***x ***(denoted ***y***_*p*_) has the length ||***y***||·cos(θ_*xy*_). Vector ***y***_*p *_lies in the same direction as vector ***x ***and may therefore be expressed as a multiple of x: ***y***_*p *_= *b*_*X*_***x***, where *b*_*X *_= (||***y***||/||***x***||)cos(θ_*xy*_) – the simple regression coefficient for *X *when *Y *is regressed on *X*. (b) If θ_*xy *_= 90° (i.e. π/2 radians), then ***x ***and ***y ***are orthogonal (denoted ***x ***⊥ ***y***), the correlation between *X *and *Y *is zero: ρ_*XY *_= cos(90°) = cos(π/2) = 0 and the regression coefficient for *Y *regressed on *X *is also zero.

When variables are represented as scaled vectors, the correlation between the original variables equates to the cosine of the angle between their corresponding vectors. Furthermore, the simple regression coefficient of one variable (the dependent variable) regressed on the other variable (the covariate) is equivalent to the orthogonal projection of the first vector on the second, i.e. a line perpendicular to the second vector is drawn from the end of the first vector, and the intersection of the line with the second vector determines the length and direction of the projection of the first vector onto the second vector. For instance, for two variables *X *and *Y *represented by vectors ***x ***and ***y***, their correlation coefficient (ρ_*xy*_) is given by cos(θ_*xy*_) where θ_*xy *_is the angle between ***x ***and ***y ***– see Figure 1. The *simple *regression coefficient of the variable *X*(*b*_*X*_), when *Y *is regressed on *X*, is the length of the perpendicular projection of ***y ***on ***x ***divided by the length of ***x ***(denoted ||***x***||), i.e. *b*_*X *_= (||***y***||/||***x***||)cos(θ_*xy*_) – see Figure 1.

### Multiple regression

**Figure 2:**
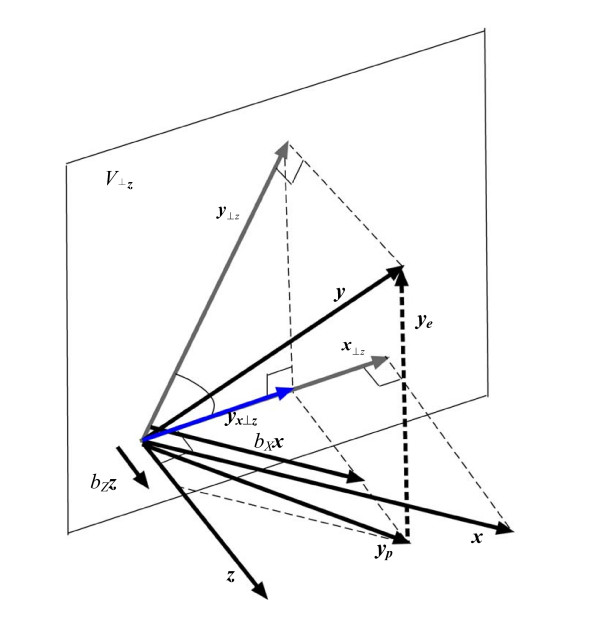
The projection of ***y***(***y***_*p*_) onto the plane spanned by ***x ***and ***z ***comprises the appropriate proportions of the vectors ***x ***and ***z ***where the proportion of vector ***x ***is *b*_*X *_and the proportion of ***z ***is *b*_*Z*_. These proportions are derived by means of the parallelogram rule: ***y***_*p *_is projected onto ***x ***parallel to the direction of ***z ***to obtain the proportion *b*_*X *_of ***x***. Likewise, **y**_*p *_is projected onto ***z ***parallel to the direction of ***x ***to obtain the proportion *b*_*Z *_of ***z***. The *P*-value for the partial regression coefficient of *X*(*b*_*X*_), when *Y *is regressed on *X *whilst also adjusting for *Z*, is derived within vector geometry from the projection of ***y ***and ***x ***onto the subspace perpendicular to ***z ***(***V***_*⊥z*_).

Regressing variable *Y *on the two variables *X *and *Z *is equivalent, within vector geometry, to finding the orthogonal projection of the vector ***y ***onto the plane spanned by the vectors ***x ***and ***z***, then using the parallelogram rule to find the contributing proportions of ***x ***and ***z ***that yield the projected vector ***y***_*p*_. For instance, if we denote the regression equation for these variables as: Y = *b*_*X*_*X*+*b*_*Z*_*Z*, where *b*_*X *_and *b*_*Z *_are partial regression coefficients, then using vector geometry: ***y***_*p *_= *b*_*X*_***x***+*b*_*Z*_***z***, where *b*_*X *_and *b*_*Z *_are the proportions (i.e. the projection weights) of the vectors ***x ***and ***z ***that make up ***y***_*p *_– see Figure 2.

Within vector geometry, the *P*-value for partial regression coefficients obtained when controlling for other covariates is derived from the projection of vectors for the outcome and each covariate onto the subspace perpendicular to all other covariates. For instance, when regressing *Y *on both *X *and *Z*, the *P*-value for the partial regression coefficient for *X *is derived from the projection of ***x ***and ***y ***onto the subspace perpendicular to ***z***. Since the entire model space is only three dimensions (spanned by ***x***, ***y***, and ***z***), the subspace perpendicular to ***z ***is a plane, denoted ***V***_*⊥z *_– see Figure 2. The *P*-value derived from the *F *ratio test for the partial regression coefficient *b*_*X *_is given as [15]:

F(1,n−3)=‖yx⊥z‖2‖ye‖2/(n−3),
 MathType@MTEF@5@5@+=feaafiart1ev1aaatCvAUfKttLearuWrP9MDH5MBPbIqV92AaeXatLxBI9gBaebbnrfifHhDYfgasaacH8akY=wiFfYdH8Gipec8Eeeu0xXdbba9frFj0=OqFfea0dXdd9vqai=hGuQ8kuc9pgc9s8qqaq=dirpe0xb9q8qiLsFr0=vr0=vr0dc8meaabaqaciaacaGaaeqabaqabeGadaaakeaacqWGgbGrdaWgaaWcbaGaeiikaGIaeGymaeJaeiilaWIaemOBa4MaeyOeI0IaeG4mamJaeiykaKcabeaakiabg2da9maalaaabaWaauWaceaaieWacqWF5bqEdaWgaaWcbaGaemiEaGNaeyyPI4LaemOEaOhabeaaaOGaayzcSlaawQa7amaaCaaaleqabaGaeGOmaidaaaGcbaWaauWaceaacqWF5bqEdaWgaaWcbaGaemyzaugabeaaaOGaayzcSlaawQa7amaaCaaaleqabaGaeGOmaidaaOGaei4la8IaeiikaGIaemOBa4MaeyOeI0IaeG4mamJaeiykaKcaaiabcYcaSaaa@4E97@

where ***y***_*x*⊥*z *_is the projection of ***y***_⊥*z *_on ***x***_⊥*z *_(which is equivalent to the projection of ***y ***on ***x***_⊥*z*_), and *n *is the sample size [16]. The value of the *F *test with 1 and *n*-3 degrees of freedom is equivalent to that of the *t*-test with *n*-3 degrees of freedom. A detailed explanation can be found in Wickens' excellent book [15].

## A geometrical illustration of adjustment for current weight in DOHaD

When examining the relationship between birth weight and disease in later life, most studies have found that the correlation and (simple) regression coefficients between the two are close to zero or slightly negative [5]. However, taking hypertension as an example, when blood pressure is simultaneously regressed on birth weight and current weight, the adjustment for current weight tends to reduce or reverse any positive association between birth weight and blood pressure, and accentuate any existing negative association between the two [17,18]. This is an effect known as the 'reversal paradox' [11].

**Figure 3:**
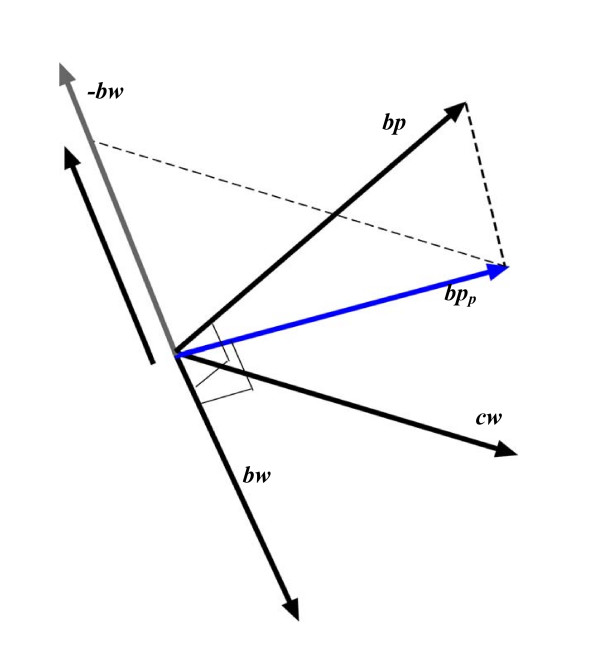
A geometrical illustration of multiple regression for blood pressure (*BP*, represented by vector ***bp***), regressed simultaneously on birth weight (*BW*, represented by vector ***bw***) and current weight (*CW*, represented by vector ***cw***).

To illustrate this geometrically, blood pressure, birth weight and current weight can be represented as vectors, ***bp***, ***bw ***and ***cw ***respectively, where the correlation between blood pressure and birth weight is nearly zero (*corr*(*BP*, *BW*) ≈ 0) – hence ***bp ***and ***bw ***are almost orthogonal. Assuming that ***bp ***and ***bw ***are orthogonal, the projection of ***bp***(***bp***_*p*_) on the plane spanned by ***bw ***and ***cw ***is also orthogonal to ***bw ***– see Figure 3. Within the multiple regression model *BP *= *b*_*BW*_*BW *+ *b*_*CW*_*CW*, the partial regression coefficient for birth weight (*b*_*BW*_) can be derived using the parallelogram rule by projecting ***bp***_*p *_onto the vector ***bw ***parallel to the direction of ***cw ***– see Figure 3. Consequently, *b*_*BW *_is not zero, but negative, due to the positive angles between ***bp ***and ***cw ***and between ***bw ***and ***cw***. In terms of variables, *b*_*BW *_is negative due to the positive correlations between blood pressure (*BP*) and birth weight (*BW*), and between current weight (*CW*) and birth weight (*BW*).

In general, the smaller the angle between ***bp ***and ***cw***, and the smaller the angle between ***bw ***and ***cw***, the greater the length of the projection of ***bp***_*p *_on ***bw ***along the vector ***cw***. In other words, the greater the positive correlation between blood pressure and current weight, and the greater the positive correlation between birth weight and current weight, the greater the absolute value of *b*_*BW*_. Moreover, while the partial regression coefficient for birth weight (*b*_*BW*_) is not zero when blood pressure is simultaneously regressed on birth weight and current weight, birth weight nevertheless contributes nothing to 'explaining' the variance in blood pressure. This is because birth weight and blood pressure are uncorrelated – i.e. they are orthogonal in vector space.

## A geometrical illustration of adjustment for weight gain in DOHaD

Since weight gain (*WG*) can be defined as the change in body weight from birth to the current time (i.e. current weight, *CW*, less birth weight, *BW*), all three variables are mathematically related such that each can be derived from the other two. Within vector geometry, this mathematical relationship means that the three vectors representing the three variables (***bw***, ***cw ***and ***wg***) span only *two *dimensions (i.e. a plane). In statistical terminology, the three variables are *collinear*, and consequently only two (not all three) can be entered simultaneously as covariates within multiple regression analyses.

**Figure 4:**
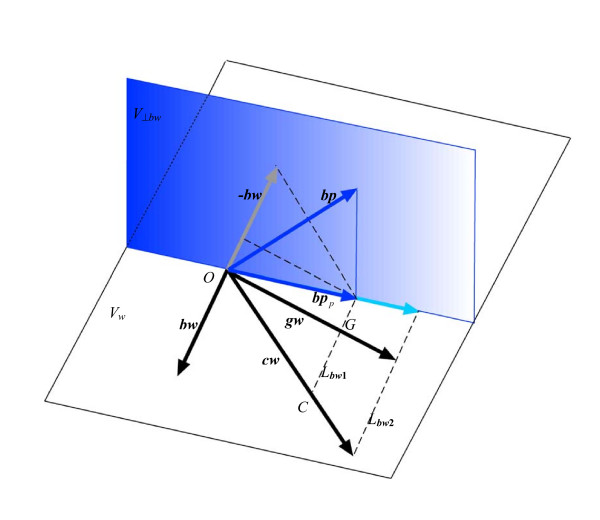
Model 1 includes birth weight (***bw***) and current weight (***cw***) as covariates; Model 2 includes birth weight (***bw***) and weight gain (***wg***) as covariates. In determining the partial regression coefficients, blood pressure (***bp***) is projected (***bp***_*p*_) onto the plane (***V***_*w*_) spanned by ***bw***, ***cw ***and ***wg***. The point *O *is the origin of the vectors ***bp***, ***bp***_*p*_, ***bw***, ***cw ***and ***wg***; *C *and *G *are the intersections of the line ***L***_*bw*1 _(running from the end of ***bp***_*p *_parallel to ***bw***) with vectors ***cw ***and ***wg***. The line ***L***_*bw*2_, crossing the tips of ***cw ***and ***wg***, runs parallel to ***L***_*bw*1_. From the two lines, ***L***_*bw*1 _and ***L***_*bw*2 _running parallel to ***bw***, it is apparent that the absolute values of the two partial regression coefficients for current weight (*CW*) and weight gain (*WG*) are identical.

From a geometrical perspective, the equivalent to regressing blood pressure simultaneously on all three covariates would be to project the vector for blood pressure (***bp***) onto the plane spanned by the three vectors representing birth weight (***bw***), current weight (***cw***), and weight gain (***wg***). However, it is impossible to assess the length of the projection ***bp***_*p *_to determine partial regression coefficients using the parallelogram rule, because the direction of this projection onto any one of the three covariate vectors (***bw***, ***cw***, or ***wg***) is now parallel to the direction of the plane spanned by the other two vectors. This dilemma results from these three covariates being multicollinear, and it can only be avoided by discarding one of the three vectors involved – equivalent to removing the corresponding variable from the regression model. Indeed, partial regression coefficients may only be determined for just two of the three covariates, since the space spanned by all three variables is only two-dimensional. Moreover, no matter which two covariates are chosen, the subspace upon which the outcome ***bp ***is projected remains the same: it is the plane ***V***_*w*_, spanned by ***bw***, ***cw ***and ***wg***. For this reason, in all three of the possible regression models – where *BP *is regressed on: (i) *BW *and *CW*; (ii) *WG *and *BW*; or (iii) *CW *and *GW *– the ***bp***_*p *_projections are identical, as are the *R*^2 ^values – see Figure 4.

To illustrate this situation, compare the following two models:

*BP *= *b*_11 _+ *b*_12_*BW *+ *b*_13_*CW *+ ε_1_;     (Model 1)

*BP *= *b*_21 _+ *b*_22_*BW *+ *b*_23_*WG *+ ε_2_;     (Model 2)

where: in Model 1, blood pressure (*BP*) is regressed on birth weight (*BW*) and current weight (*CW*), with *b*_11_, *b*_12_, *b*_13_, and ε_1 _being the model intercept, partial regression coefficients for birth weight and current weight, and the residual error, respectively; in Model 2, current weight is replaced by weight gain (*WG*) and the regression coefficients and residual error are now *b*_21_, *b*_22_, *b*_23 _and ε_2_, respectively. Despite these differences, for the reasons mentioned earlier, these two models have the same degree of fit (*R*^2^), and the residuals of both are identical (ε_1 _= ε_2_).

Using the parallelogram rule to derive partial regression coefficients for each model, consider the line ***L***_*bw*1_, which runs parallel to ***bw ***from the tip of ***bp***_*p *_to intersect ***cw ***and ***wg ***– see Figure 4. The partial regression coefficient *b*_13 _for *CW *in Model 1 is the length of ***cw ***intersected by ***L***_*bw*1_, i.e. the length of the vector ***OC ***divided by the length of ***cw***. Similarly, the partial regression coefficient *b*_23 _for *WG *in Model 2 is the length of the vector ***OG ***divided by the length of ***wg***. Since ***cw ***= ***bw ***+ ***wg***, the line ***L***_*bw*2 _is parallel to ***L***_*bw*1 _and, by elementary trigonometry, the ratio of the lengths of ***OC ***and ***cw ***is identical to the ratio of the lengths of ***OG ***and ***wg ***– see Figure 4. Therefore, although birth weight has different partial regression coefficients in each model (*b*_12 _≠ *b*_22_), the partial regression coefficients for current weight in Model 1 and weight gain in Model 2 are identical (*b*_13 _= *b*_23_).

When using vector geometry to determine the *P*-value for the partial regression coefficient of current weight in Model 1 or weight gain in Model 2, whilst adjusting for birth weight, it is necessary to identify the corresponding vector subspace perpendicular to ***bw***. This is the same vector subspace for each model and is a plane, denoted ***V***_*⊥bw *_– see Figure 4. Thus, the partial regression coefficient *P*-value for current weight in Model 1 is derived by projecting ***bp ***and ***cw ***onto ***V***_*⊥bw*_. Similarly, the partial regression coefficient *P*-value for weight gain in Model 2 is derived by projecting ***bp ***and ***wg***onto ***V***_*⊥bw*_. Since ***cw ***= ***bw ***+ ***wg***, the projection of ***cw ***or ***wg ***onto ***V***_*⊥bw *_is identical, albeit in the reverse direction to ***bw ***– see Figure 4. Consequently, regressing blood pressure on either current weight or weight gain, whilst also adjusting for birth weight, yields identical partial regression coefficient *P*-values for current weight in Model 1 and weight gain in Model 2.

In general, when birth weight is a covariate in multiple regression together with current weight, or any variable that is a linear combination of birth weight and current weight (such as weight gain), the partial regression coefficients for either of these will be identical in magnitude, as will their respective *P*-values – even though the direction of the coefficients will depend on the nature of the linear relationship concerned.

Finally, we now consider a third model, where blood pressure (*BP*) is simultaneously regressed on weight gain (*WG*) and current weight (*CW*):

*BP *= *b*_31 _+ *b*_32_*WG *+ *b*_33_*CW *+ ε_3_;     (Model 3)

where *b*_31_, *b*_32_, *b*_33 _and ε_3 _are the intercept, the partial regression coefficients for weight gain and current weight, and the residual error, respectively. We know that Models 1 to 3 have identical *R*^2 ^values and identical residuals (i.e. ε_1 _= ε_2 _= ε_3_). Furthermore, it can be shown that the partial regression coefficients for birth weight in Model 1 and weight gain in Model 3 are identical (*b*_32 _= -*b*_12_) with identical *P*-values. Thus, when current weight is a covariate in multiple regression analyses together with either birth weight or weight gain, the absolute partial regression coefficients for birth weight and weight gain are identical, as are their *P*-values, model fit and, hence, the proportion of variance explained.

## Discussion

Lucas et al. [9] have previously discussed the algebraic relationship of regression coefficients amongst the three multivariable models presented above. However, in this article, we used vector geometry to demonstrate why these three models are effectively equivalent. Not only do the partial regression coefficients exhibit algebraic relationships, but the coefficient *P*-values and the variances explained are identical. The crucial issue, therefore, remains the interpretation of these models. For instance, when adjusting for birth weight it is impossible to differentiate between the effects of current weight or weight gain on blood pressure, since either covariate gives rise to identical coefficient *P*-values and an equivalent proportion of outcome variance explained. Conversely, when adjusting for current weight, the impact of weight gain on blood pressure is identical to that of birth weight, albeit in the opposite direction. For these reasons, the apparent finding that weight gain has an 'independent' statistical relationship with blood pressure may not reflect any genuine aetiological relationship.

From a clinical viewpoint, higher weight gain is equivalent to higher current weight if one adjusts for birth weight (i.e. holds birth weight constant). Under these circumstances, arguing that weight gain is related to blood pressure is equivalent to arguing that current weight is related to blood pressure, which we know to be true. Furthermore, while adjusting for current weight tends to create a stronger inverse relationship between birth weight and blood pressure, it will also strengthen the positive relationship between blood pressure and current weight. It is therefore unclear whether it is current weight or weight gain that contributes to elevated blood pressure, or both. Indeed, the stronger relationship between current weight and blood pressure after adjusting for birth weight might be interpreted as either: (i) that the impact of weight is cumulative and linear; or (ii) that heavier people also have, on average, larger birth weights. In the latter scenario, adjusting for birth weight would be interpreted as removing its 'protective' effect on blood pressure, thereby increasing the strength of its relationship with current weight. An alternative interpretation of the same regression model would be that, holding current weight constant, those with greater weight gain must have a lower birth weight, and hence the greater the weight gain the higher the blood pressure.

## Conclusion

As we have seen using vector geometry, weight gain can be a proxy for either current weight (by adjusting for birth weight) or birth weight (by adjusting for current weight). Consequently, the role of weight gain in many of the regression models commonly adopted to compare the pre- and post-natal developmental origins of health and disease is essentially ambiguous.

## Competing interests

The author(s) declare that they have no competing interests.

## Authors' contributions

YKT developed the idea of using vector geometry. All authors contributed to drafting and editing the manuscript.

## Appendix

### Basic geometric tools

The most common form of geometry in clinical research occurs in what is termed *variable space*, illustrated for instance by a scatter plot. In the scatter plot of two variables, say *X *and *Y*, each with *n *independent observations (*X*_1 _... *X*_*n*_) and (*Y*_1 _... *Y*_*n*_), there will be *n *points in 2-dimensional space (i.e. on a plane). The axes represent variables *X *and *Y*, and the points are the observations made on each subject. In place of using variables as axes, the same data may be displayed in what is termed 'subject space', using subjects as the axes (of which there would now be *n*) and the variables *X *and *Y *become two points (in *n*-dimensional space). By connecting the origin with each point, *X *and *Y *become vectors in *n*-dimensional space, with coordinates (*X*_1 _... *X*_*n*_) and (*Y*_1 _... *Y*_*n*_) respectively.

**Figure 6:**
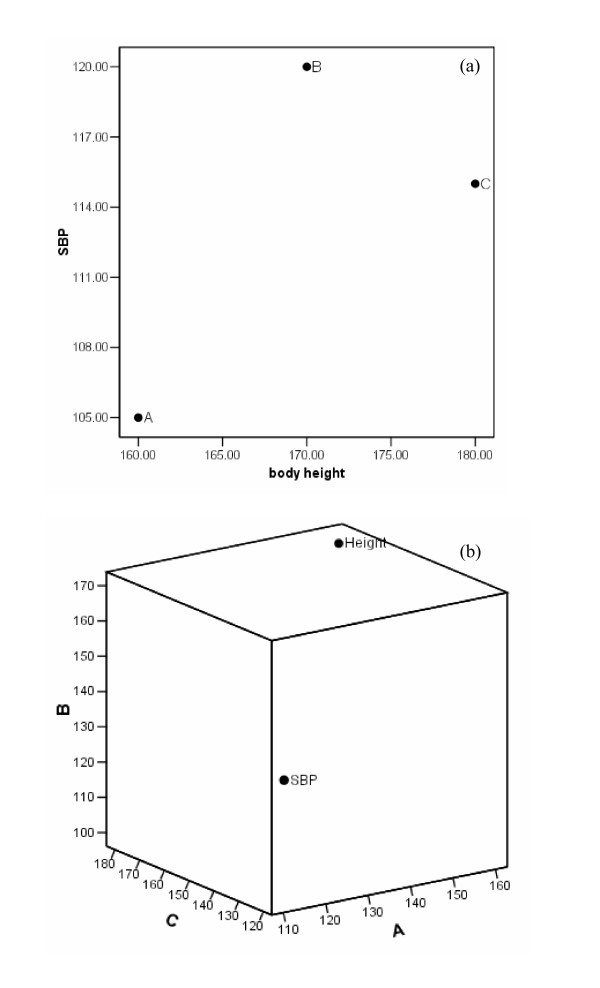
(a) The two-dimensional plot of three subjects with measurements of two variables: systolic blood pressure [SBP] and body height in variable space; (b) the three-dimensional plot of the same data in subject space.

Figure 6a and 6b illustrate the difference between variable and subject space using a numerical example. Suppose the body height and systolic blood pressure of three subjects **A**, **B **and **C **are measured. In variable space, the data are displayed as three points representing the three subjects in a two-dimensional scatter plot (Figure 6a). In contrast, in subject space, the data are displayed as two points representing the two variables in a three-dimensional scatter plot (Figure 6b).

Although it is impossible to visualize *n*-dimensional space, we only need two dimensions (i.e. a plane) to visualize the relative relationship between the two vectors representing *X *and *Y*. We effectively 'drop' the original axes, retaining only the relative relationship between the vectors representing the variables. In general, the number of dimensions needed to represent variables in subject space is no greater than the number of variables. Whilst it remains impossible to visualize four or more dimensions, using this condensed form of vector space, only two dimensions are required to illustrate the principles of simple regression, and only three dimensions are required to illustrate the principles of multiple regression. It is therefore useful to represent the original variable, e.g. *X*, as scaled vector, ***x***, where each original data point, *X*_*i*_, is transformed to *x*_*i *_such that the length of the vector (||***x***||) is equal to the standard deviation (SD) of the original variable. This is achieved using the following formula:

xi=[Xi−(∑i=1nXin)]n−1.     (Eq.A1)
 MathType@MTEF@5@5@+=feaafiart1ev1aaatCvAUfKttLearuWrP9MDH5MBPbIqV92AaeXatLxBI9gBaebbnrfifHhDYfgasaacH8akY=wiFfYdH8Gipec8Eeeu0xXdbba9frFj0=OqFfea0dXdd9vqai=hGuQ8kuc9pgc9s8qqaq=dirpe0xb9q8qiLsFr0=vr0=vr0dc8meaabaqaciaacaGaaeqabaqabeGadaaakeaacqWG4baEdaWgaaWcbaGaemyAaKgabeaakiabg2da9maalaaabaWaamWaceaacqWGybawdaWgaaWcbaGaemyAaKgabeaakiabgkHiTmaabmGabaWaaabmaeaadaWcaaqaaiabdIfaynaaBaaaleaacqWGPbqAaeqaaaGcbaGaemOBa4gaaaWcbaGaemyAaKMaeyypa0JaeGymaedabaGaemOBa4ganiabggHiLdaakiaawIcacaGLPaaaaiaawUfacaGLDbaaaeaadaGcaaqaaiabd6gaUjabgkHiTiabigdaXaWcbeaaaaGccqGGUaGlcaWLjaGaaCzcamaabmGabaGaeeyrauKaeeyCaeNaeeOla4IaeeyqaeKaeGymaedacaGLOaGaayzkaaaaaa@4F73@

Other variables (e.g. *Y*) are similarly transformed to yield vectors (***y***). An immediate advantage of this approach is that the correlation coefficient between the variables *X *and *Y *is the cosine of the angle between the vectors ***x ***and ***y***. For instance, when the correlation between *X *and *Y *is zero, the angle between ***x ***and ***y ***is 90° (i.e. π/2 radians), and the two vectors are therefore orthogonal (denoted ***x ***⊥ ***y***). Similarly, when the correlation between *X *and *Y *is 0.5, the angle between ***x ***and ***y ***is 60° (i.e. π/3 radians). Another advantage of representing variables as scaled vectors in this way is that the number of dimensions needed for regression analyses is reduced by one. For instance, if *Y *is regressed on *X*, there are three variables in the equation: *Y*, *X *and the intercept (a vector with the value 1 for all its observations). After the transformation of Eq.A1, the intercept becomes a zero vector, and hence redundant. Therefore, we need at most *k *dimensions to represent *k *variables in *subject space *when examining the role of multiple regression.

**Figure 5:**
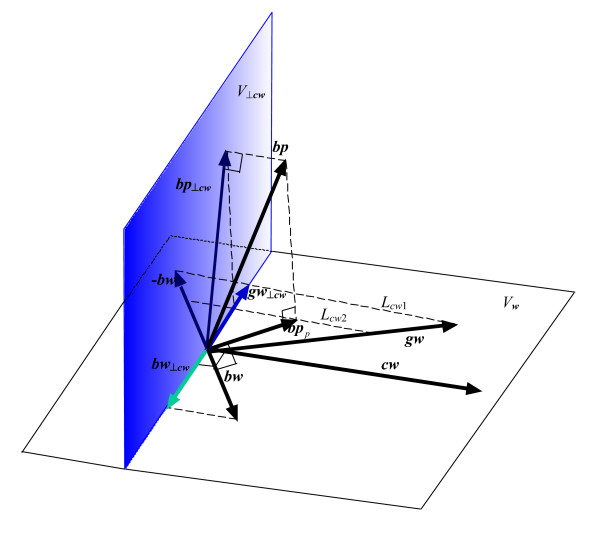
The projection of ***bp ***on ***V***_*w*_and ***V***_*⊥cw *_is vector ***bp***_*p *_and ***bp***_*⊥cw *_respectively. Since ***cw ***= ***bw ***+ ***wg***, the projections of ***bw ***and ***wg ***on ***V***_*⊥cw *_(***bw***_*⊥cw *_and ***wg***_*⊥cw *_respectively) will be in opposite directions (though parallel). Therefore, if the angle between ***bw***_*⊥cw *_and ***bp***_*⊥cw *_is φ, the angle between ***wg***_*⊥cw *_and ***bp***_*⊥cw *_will be (π - φ). From elementary trigonometry: cos(φ) = -cos(π - φ). Hence, in Model 3, after adjustment for current weight (*CW*), the *P*-value for weight gain (*WG*) is identical to that for birth weight (*BW*) in Model 1. From the two lines, ***L***_*cw*1 _and ***L***_*cw*2 _running parallel to ***cw***, it is apparent that the absolute values of the two partial regression coefficients for birth weight (*BW*) and weight gain (*WG*) are identical.
